# Prevalence and Risk Factors of Subclinical Hypothyroidism in Young and Middle-Aged Patients With First-Episode Drug-Naïve Major Depressive Disorder

**DOI:** 10.1155/da/3154096

**Published:** 2025-04-16

**Authors:** Jiacheng Liu, Liying Yang, Chuanyi Kang, Xiaohong Wang, Na Zhao, Xiangyang Zhang

**Affiliations:** ^1^Department of Psychiatry, The First Affiliated Hospital of Harbin Medical University, Harbin, Heilongjiang Province, China; ^2^Department of Psychiatry, Anhui Mental Health Center, Hefei Fourth People's Hospital, Affiliated Psychological Hospital of Anhui Medical University, Hefei, Anhui Province, China

**Keywords:** low-density lipoprotein cholesterol, major depressive disorder, middle-aged adults, subclinical hypothyroidism

## Abstract

**Background:** Subclinical hypothyroidism (SCH) is a mild impairment of thyroid function. The prevalence of SCH is significantly higher in the major depressive disorder (MDD) population than in the general population, but the risk factors and relationships are not apparent. The occurrence of SCH is influenced by age and medication. Therefore, our study was to investigate the prevalence and risk factors of SCH in young and middle-aged groupstotal of patients with first-episode and drug-naive (FEDN) MDD.

**Methods:** A total of 1717 FEDN MDD patients were divided into a younger group (18–45 years) and a middle-aged group (>45 years). The Hamilton Depression Scale (HAMD) was used to assess patients' depression symptoms. Serum thyroid function and lipid level parameters were measured. A self-administered questionnaire collected other clinical and demographic data.

**Results:** The prevalence of SCH in middle-aged MDD patients was 66.9%. Middle-aged patients had a longer duration of illness, a later age of onset, a higher proportion of female patients, and a lower level of education. Further logistic regression indicated that serum total cholestrol (TC) and high-density lipoprotein cholesterol (HDL-C) levels, as well as overweight and obesity, were significantly associated with SCH in both groups; however, low-density lipoprotein cholesterol (LDL-C) was an independent risk factor associated with SCH in the middle-aged group.

**Conclusions:** Our results suggest that the prevalence of SCH is higher in middle-aged MDD patients than in younger patients and that long-term more severe depression, high TC and HDL-C levels, and abnormal body weight may influence the occurrence of SCH. Physicians should pay more attention to LDL-C levels in middle-aged patients with FEDN MDD.

## 1. Introduction

Subclinical hypothyroidism (SCH) is defined as elevated thyroid stimulating hormone (TSH) levels with free thyroid hormone (T4) levels within the reference range [1]. More than 20% of patients with autoimmune thyroiditis experience depression each year with anti-thyroglobulin and thyroid peroxidase antibodies found to be at high risk for metabolic syndrome and suicide [2, 3]. SCH patients with major depressive disorder (MDD) [4], with prevalence estimates ranging from 9.4% to 60.6% [5, 6]. Depressed patients with SCH were seven times more likely to develop metabolic syndrome than those without SCH, and the number of components of metabolic syndrome positively correlated with SCH's prevalence [5]. Therefore, MDD patients with combined SCH should be given high priority. Recently, many studies have investigated the relationship between SCH and MDD [7, 8]. Several reports have focused on SCH and its potential neuropsychiatric and neurocognitive outcomes; however, there is no consensus on the results of these reports [8–10].

A survey of thyroid function in adults in 31 provinces in China showed that the prevalence of hypothyroidism was 13.95%. The prevalence of overt hypothyroidism (OH) and SCH was 1.02% and 13.93%, respectively. For every 10-year increase in age, the prevalence of mild SCH, severe SCH, and OH increased by 1.16%, 1.40%, and 1.29%, respectively [11]. However, there is growing evidence that the adverse health effects of thyroid dysfunction found in younger age groups cannot be extrapolated to older adults [12]. The prevalence of SCH increases with age. This may be due to changes in thyroid metabolism associated with aging, but findings are inconsistent [8, 13–16]. Serum thyroxine levels increase with age in persons without thyroid disease; serum thyrotropin concentrations in older patients may exceed the upper limit of the traditional reference range of 4–5 mU/L. This phenomenon may lead to an overestimation of the true prevalence of SCH in people over 70 years of age [1]. It has been suggested that the reasons for the change in TSH levels due to spontaneous normalization in the majority of older adults with SCH remain undisclosed [17].

Furthermore, clinical outcomes, such as cardiovascular risk [18, 19] and the ability to perform activities of daily living [20], were associated with thyroid dysfunction in younger age groups and were not replicated in older adults. Although the increased prevalence of hypothyroidism in older adults is accompanied by higher rates of physical and cognitive impairment, depression, and cardiovascular disease, hypothyroidism may have a protective effect on the health of older adults [21]. The overall impact of SCH on older adults varies, and individualized treatment and long-term monitoring are appropriate strategies [22, 23]. Although several cohort studies have evaluated the association between SCH and depression, most of these studies have focused on the elderly population, with little evidence of an association between SCH and depression in middle-aged and younger adults. In addition, older adults have complex somatic disorders that may be accompanied by multiple somatic disorders and perhaps include too many confounding factors. At the same time, it has been noted that despite the well-characterized age-related changes in thyroid function, normal TSH reference limits derived for specific age groups are not routinely used to identify thyroid dysfunction in older adults [24]. Hence, this study included younger and middle-aged adults for comparative studies.

Early and routine screening for depression is vital in preventing morbidity and mortality [25]. Studies have shown that growth inhibitors and serotonin affect the hypothalamic–pituitary–thyroid axis, which links hypothyroidism to depression [25]. SCH has a negative impact on depression. Many depressed patients have elevated antithyroid antibodies. Microsomal antibodies are also common in patients with chronic lymphocytic thyroiditis. Additional TSH receptor (TSHR) antibodies block the function of TSH, which can lead to hypothyroidism [26]. Diminished TSH response to TRH has been associated with an increased risk of suicide, suicidal ideation, violent suicide attempts, and elevated mortality [27]. Thyroid disease and mood disorders are both common and can occur in the same person, so it is important for psychiatrists to optimize the treatment of patients with these comorbidities. Optimal intervention requires an understanding of the subtle thyroid dysfunction that may be present in patients with mood disorders. Early detection and treatment of SCH may be beneficial to improve patients' quality of life and affect multiple organs.

Therefore, understanding the link between SCH and depression at the symptom level can help to diagnose and treat both disorders accurately. However, no studies have shown whether SCH is differentially associated with different depressive symptoms among different age subgroups. Therefore, the aims of this study were (1) to elucidate SCH in patients with first-episode and drug-naive (FEDN) MDD and different age groups and (2) to identify different risk factors in different age groups, which are crucial for early screening for SCH and intervention in patients with depression.

## 2. Methods

### 2.1. Subjects

All participants were recruited at the psychiatric outpatient clinic of the First Affiliated Hospital of Shanxi Medical University from 2015 to 2017. The study was approved by the Institutional Review Board (IRB) of the First Affiliated Hospital of Shanxi Medical University and was conducted in accordance with the Declaration of Helsinki (2016-Y27). After a detailed explanation, all participants or guardians signed an informed consent form.

A total of 1717 FEDN MDD participants were recruited in this cross-sectional study. All participants recruited in the study met the following inclusion criteria: (1) age between 18 and 60 years; (2) never received antidepressants or other psychotropic medications; (3) meeting the diagnosis of MDD in the Diagnostic and Statistical Manual of Mental Disorders, Fourth Edition (DSM-IV); and (4) ≥ 24 on the 17-item Hamilton Depression Scale (HAMD-17). Exclusion criteria were (1) a severe physical illness; (2) a history of drug abuse or alcohol abuse and dependance; (3) a neurological disorder, head injury, or any other illness with psychiatric features; (4) pregnant or breastfeeding women; and (5) refusal to sign an informed consent.

### 2.2. Clinical Measurements

Two trained clinical psychiatrists interviewed patients for their sociodemographic information and clinical data. The two psychiatrists with at least 5 years of clinical practice attended a training session on the use of the HAMD-17 before the start of this study. The HAMD was used to measure the severity of depressive symptoms [28], which consists of eight five-point ratings reverified as normal weight (BMI < 24 kg/m^2^), overweight (24 ≤ BMI < 28 kg/m^2^), and obese (BMI ≥ 28 kg/m^2^) [29].

### 2.3. Biomarker Measurements

In this study, FT4 values in the normal range and TSH above 4.2 mIU/L were defined as SCH, so patients were divided into two groups: with and without SCH. Biomarker samples were collected from each participant from 6:00 a.m. to 8:00 a.m., then centrifuged and stored at −20°C for testing. An electrochemiluminescent immunoassay was used to determine the concentration of thyroid function in serum. An enzymatic colorimetric assay was used to detect lipid profiles.

The measured parameters in this study included total cholesterol (TC)(Marangell LB), triglycerides (TG), high-density lipoprotein cholesterol (HDL-C), and low-density lipoprotein cholesterol (LDL-C), as well as thyroid function, TSH, free triiodothyronine (FT3), free thyroxine (FT4), thyroglobulin antibody (A-TG), and thyroid peroxidase antibody (A-TPO).

### 2.4. Statistical Analysis

In this study, SPSS 23.0 software was used for data analysis. Mean ± standard deviation or percentages were used to describe the data. Demographic and clinical data were analyzed by ANOVA and chi-square test for continuous and categorical variables, respectively. Bonferroni correction was used to correct multiple tests. To compare age differences in SCH and clinical data, we used a 2 × 2 ANOVA, considering diagnosis (with and without SCH) and age (young and middle-aged patients). We then used Pearson correlation coefficients to assess the association between SCH and other clinical variables. Finally, binary logistic regression was performed to analyze the independent risk factors affecting SCH in young and middle-aged patients with MDD. A binary logistic regression model was established based on univariate analysis of the two comparison groups (young vs. elderly), with the presence or absence of SCH as the dependent variable.

## 3. Results

### 3.1. Clinical Characteristics of Young and Middle-Aged MDD Patients With and Without SCH

Based on age, participants were divided into a younger group (aged ≤45 years) and a middle-aged group (>45 years).  Table 1 shows the clinical characteristics of young and middle-aged MDD patients in the SCH and no-SCH groups. In the younger group, the prevalence of SCH was 58.3% (751/1288), while in the middle-aged group, the prevalence of SCH was 66.9% (287/429). The prevalence of SCH was significantly higher in the middle-aged group than in the young group (*χ*^2^ = 9.938, *p*=0.002). Compared to the no-SCH group, the SCH group had a longer duration of illness (*F* = 62.830, *p* < 0.001), higher HAMD score (*F* = 196.659, *p* < 0.001), higher TC (*F* = 288.487, *p* < 0.001), TG (*F* = 12.668, *p* < 0.001) and LDL-C levels (*F* = 154.913, *p* < 0.001), greater prevalence of overweight and obesity (*χ*^2^ = 70.342, *p* < 0.001), and lower HDL-C levels (*F* = 84.214, *p* < 0.001).

Compared to younger patients, middle-aged patients had a longer duration of illness (*F* = 70.114, *p* < 0.001), a later age of onset (*F* = 2442.107, *p* < 0.001), a higher prevalence in women (*χ*^2^ = 11.537, *p*=0.001), and fewer years of education (*χ*^2^ = 302.224, *p* < 0.001). They all passed the Bonferroni correction (*p* < 0.05/10 = 0.005). In addition, SCH х age also significantly affected the duration of illness (*F* = 12.813, *p* < 0.001).

### 3.2. Relationship Between SCH and Clinical Data in Young and Middle-Aged MDD Patients

The results of the relationship between SCH and clinical data in the young and middle-aged groups are shown in Table 2. In the young group, SCH was associated with duration of illness (*r* = 0.189, *p* < 0.001), HAMD score (*r* = 0.421, *p* < 0.001), TC (*r* = 0.463, *p* < 0.001), HDL-C (*r* = −0.249, *p* < 0.001), TG (*r* = 0.110, *p* < 0.001), LDL-C (*r* = 0.341, *p* < 0.001), and overweight/obesity (*r* = 0.185, *p* < 0.001).

In the middle-aged group, SCH was associated with duration of illness (*r* = 0.308, *p* < 0.001), HAMD score (*r* = 0.333, *p* < 0.001), TC (*r* = 0.453, *p* < 0.001), HDL-C (*r* = −0.248, *p* < 0.001), TG (*r* = 0.096, *p*=0.047), LDL-C (*r* = 0.375, *p* < 0.001), and overweight/obesity (*r* = 0.245, *p* < 0.001). However, the association between SCH and TG levels did not pass the Bonferroni correction (*p* < 0.05/10 = 0.005).

### 3.3. Demographic and Clinical Variables Independently Associated With SCH in Each Age Group

The results of independent factors associated with SCH in each group are shown in Table 3. In the younger group, duration of illness (OR = 1.070, 95% CI = 1.034–1.107, *p* < 0.001), HAMD score (OR = 1.231, 95% CI = 1.162–1.303, *p* < 0.001), TC (OR = 1.921, 95% CI = 1.585–2.327, *p* < 0.001), HDL-C (OR = 0.154, 95% CI = 0.091–0.260, *p* < 0.001), overweight (OR = 2.135, 95% CI = 1.623–2.809, *p* < 0.001), and obesity (OR = 4.096, 95% CI = 1.807–9.258, *p*=0.001) were all independent risk factors associated with SCH.

In the middle-aged group, duration of illness (OR = 1.079, 95% CI = 1.030–1.130, *p*=0.001), HAMD score (OR = 1.107, 95% CI = 1.006–1.217, *p*=0.036), TC (OR = 1.885, 95% CI = 1.346–2.641, *p* < 0.001), HDL-C (OR = 0.297, 95% CI = 0.123–0.716, *p*=0.007), LDL-C (OR = 1.639, 95% CI = 1.121–2.398, *p*=0.011), overweight (OR = 3.354, 95% CI = 2.023–5.561, *p* < 0.001), and obesity (OR = 21.695, 95% CI = 2.020–233.056, *p*=0.011) were all independent risk factors associated with SCH.

## 4. Discussion

To our knowledge, this is the first study to examine the differences in factors influencing the development of SCH in young and middle-aged MDD patients. We found that middle-aged SCH patients had a longer duration of illness, a later onset, a higher proportion of female patients, and a lower level of education than young SCH patients. Duration and severity of depression, serum TC and HDL-C levels, and overweight and obesity all influenced the development of SCH in young and middle-aged MDD patients. In addition, LDL-C levels may predict the emergence of SCH in middle-aged MDD patients.

Our study found that the prevalence of SCH was 66.9% in the middle-aged MDD group, significantly higher than that of 58.3% in the young group. Previous studies have shown that the prevalence of SCH is much higher in patients with depression compared to the general population. More than 63% of SCH patients reported depressive symptoms [30, 31]. There may be several reasons for the inconsistent results of previous studies. First, in depressed patients, antidepressants or antipsychotics affect metabolism, leading to adverse effects, such as weight gain and dyslipidemia. The patients, included in our study, were first-episode unmedicated patients. Second, depression, thyroid disease, and lipid metabolism may have the same pathogenesis and interact with each other, thus affecting the prevalence rate. In addition, the diagnostic criteria for SCH are varied, which contributes to its different prevalence rates. Age has been suggested as an interactive factor in the correlation between depressive symptoms and FT3, TT3, TT4, TPOAb, and TG [32]. One study noted that for every one-quarter increase in TSH, both free T3 (FT3) and FT3:FT4 ratio increase, while FT4 decreased significantly. However, as part of the aging process, the conversion of T4 to T3 decreases with age [33]. As a result, TSH changes significantly with age, and the prevalence of SCH also changes significantly with age.

In our study, we found that middle-aged MDD patients presenting with SCH had a later onset and lower education level than younger patients. Because of the rapid development of society and the pressure of life, young people face greater pressure, so the age of onset of the disease is a little earlier; and because young people have a higher level of education, the media develops faster, and there is better dissemination of the disease, young people can find out the abnormalities of the thyroid function more quickly. We also found a higher prevalence in women in the middle-aged SCH group compared to the younger group. Gender has also been identified as a factor in the interaction between depressive symptoms and FT3, TT3, and TSH [32]. Basal thyroid hormone concentrations tend to increase, especially in women, whereas nocturnal peaks in basal thyroid hormone and decreases in thyroid hormone release with age are more common in men [34]. In a screening of 10,405 individuals, SCH was found to be more common in women (2.4% in men and 5.8% in women, *p* < 0.001) and to increase with age (*p* < 0.001). The high prevalence of autoimmune thyroid disease in women may also contribute to the higher incidence of SCH in women over 45 years of age [35].

We explored the factors influencing the onset of SCH in young and middle-aged adults. We found that the duration and severity of depression impacted the onset of SCH, as previous studies found a higher prevalence of depressive symptoms and worse scores on the depression scale in patients with SCH [36, 37]. A recent study from the Shanghai Mental Health Center in China noted that thyroid function has a greater impact on the risk of readmission after discharge in patients with MDD and that the longer the duration of illness in patients with MDD, the greater the likelihood of SCH [38]. Previous studies have noted that the severity of MDD significantly affects serum TSH levels. This may be due to the fact that TSH is involved in mood regulation in two ways, one acting on alpha and beta subtypes of thyroid hormone receptors in the limbic system and the other regulating 5-TH and GABA in the brain [39]. Our findings suggest that we should pay more attention to the development of SCH in depressed patients with longer duration and more severe depression.

In addition, we found that overweight and obesity influenced the development of SCH. A national study showed that obesity is a risk factor for SCH and thyroid autoimmunity [40]. Thyroid hormones play a crucial role in regulating metabolism. They affect the balance between metabolic rate and caloric intake, affecting weight changes. SCH is associated with obesity; almost a quarter of patients are obese [41]. Appetite and weight disturbances are common in depressive syndromes. Resting metabolic rate may also be altered in depressed patients. In large population studies, serum TSH is usually positively correlated with body weight and body mass index, and elevated TSH may contribute to overweight or obesity, an effect that may be mediated in part by leptin [42]. Conversely, it has been suggested that centrally obese individuals have a higher prevalence of thyroid dysfunction and depression compared to nonobese individuals. Several studies have also reported that obese individuals have higher serum TSH concentrations and lower FT4 levels than nonobese individuals. The main mechanism responsible for this difference is thought to be increased thyroxine 5-deiodinase activity in obese patients. The local conversion of T4 to T3 by thyroxine 5-deiodinase is a key mechanism in thyroid hormone regulation [43].

Our findings also showed that TC and HDL-C levels were independent influencers of SCH in middle-aged and young depressed patients. Many studies support our results. For example, Duntas and Brenta [44] found higher TC levels and lower HDL-C levels in SCH patients with TSH >10 μL. A recent study showed a significant correlation between hypothyroidism and hypertriglyceridemia, and SCH was associated with a significantly higher risk of hypertriglyceridemia and high LDL-C [45]. Another study showed that TC increased with TSH, and HDL-C decreased with TSH, even in individuals with normal thyroid function [46]. This may be due to the fact that MDD and thyroid disease may share a common etiology—an imbalance in the neuroendocrine network. There is an evidence that the pathologic development of hypothyroidism-associated hyperlipidemia is associated with the downregulation of thyroid hormones and upregulation of TSH in serum [47].

Interestingly, we found that LDL-C levels were an independent influencing factor on the development of SCH in the middle-aged group but not in the younger group. A large meta-analysis shows that SCH is associated with a high risk of atherosclerosis [48]. It is well-known that advanced age is one of the most potent independent risk factors for the development of atherosclerosis. The prevalence of hypercholesterolemia and high LDL-C levels increases progressively with age, reaching a peak in the 55–64 years age group [49]. Another study showed a positive correlation between hydroxy-octadecadienoic acids in LDL and TSH [50]. All these studies show the correlation between LDL-C levels and SCH, suggesting that LDL-C may influence the development of SCH in the elderly population. A possible reason for this is the correlation of cholesterol with TSH and cerebral glucose metabolism (CMglu). One study found that serum TSH levels were negatively correlated with CMglu in euthyroid patients with mood disorders [51]. Serum lipid abnormalities may be the leading cause mediating the increase in TSH and decrease in CMglu, as SCH may lead to elevated serum cholesterol levels, which are associated with a decrease in CMglu in various brain regions, including the precuneus, in late middle age [52].

The innovation of this paper is that since thyroid function in elderly patients is affected by many factors and is associated with many somatic diseases, this paper focuses on comparing the prevalence of SCH and risk factors for its development in young and middle-aged patients with MDD. Meanwhile, in order to avoid the effect of medication on thyroid function, we selected patients with first-episode and drug-naïve MDD. The intention was to explore more preventive and diagnostic ideas for young and middle-aged patients.

However, this study also has some limitations. First, this study was cross-sectional; some data were from observational studies. Our findings support the hypothesis that there are differences in factors influencing SCH in young and middle-aged MDD patients. Also, a longitudinal study is needed to confirm these results. Second, we did not consider other hematological indicators that may also impact depression, like inflammatory factors, white blood cells, cortisol, serotonin, etc. Third, we measured TSH and free T4 only at a single time point at baseline, the diagnosis of SCH is usually based on a single assessment of TSH without at least two validated measurements. In the future, we will conduct a more comprehensive assessment of thyroid function in enrolled patients and avoid bias through multi-nodal sampling and the addition of controlled trials. This approach may lead to the potential misclassification of typical thyroid participants with transiently elevated TSH levels. Fourth, this study only compared the prevalence of SCH and the risk factors for its development in young and middle-aged patients with FEDN MDD. In the future, we will be more detailed in age division to better illustrate the pattern of SCH development in patients with MDD. Finally, in this study, we focused on the factors that influence the presence of SCH in patients of different age groups. We employed binary logistic regression to examine the independent effects of various predictors, focusing primarily on their respective contributions. Although we have not explored the interactions between these factors, future studies will investigate this aspect. Specifically, we plan to include TSH as a variable, along with other relevant covariates, to assess potential interactions using more complex analytical methods, such as multiple logistic regression and mediation analysis. The above analysis will allow us to explore the magnitude of influence and the interplay between different factors in a more comprehensive manner, thereby enhancing the depth and scope of our findings.

In conclusion, this study suggests that middle-aged MDD patients are at higher risk of developing SCH compared with younger MDD patients. Long-term, more severe depression, elevated TC and HDL-C levels, and abnormal body weight may independently contribute to the occurrence of SCH. Furthermore, these factors may interact with thyroid function, and the risk may be exacerbated by chronic severe depression, abnormal eating habits, and metabolic abnormalities. Physicians should pay particular attention to LDL-C levels in middle-aged MDD patients, as they may play a unique role in the development of SCH. Regular screening of serum lipid levels and thyroid function is recommended to prevent the development of SCH in patients with MDD. These findings provide valuable insights for healthcare providers to assess the risk of SCH in MDD patients and highlight the need for comprehensive monitoring of metabolic and thyroid health in this population.

## Figures and Tables

**Table 1:** Clinical characteristics of young and middle-aged MDD patients with and without SCH (*n* = 1717).

Variables	MDD without SCH		MDD with SCH		SCH *F*/*χ*^2^ (*p*)	Age *F*/*χ*^2^ (*p*)	SCH × age *F*/*χ*^2^ (*p*)
	Young	Middle-aged	Young	Middle-aged			
Sample size	537	142	751	287	—	—	—
Duration of illness	4.98 (3.97)	6.25 (5.35)	6.13 (4.12)	9.29 (5.82)	62.830 (<0.001)	70.114 (<0.001)	12.813 (<0.001)
Age of onset	28.64 (8.52)	51.82 (4.37)	29.47 (8.63)	51.00 (4.07)	0.000 (0.993)	2442.107 (<0.001)	3.349 (0.067)
HAMD	28.77 (2.63)	29.29 (2.93)	31.19 (2.69)	31.31 (2.79)	196.659 (<0.001)	4.072 (0.044)	1.583 (0.209)
TC	4.62 (0.90)	4.75 (0.89)	5.61 (1.04)	5.74 (1.06)	288.487 (<0.001)	4.730 (0.030)	0.008 (0.928)
HDL-C	1.37 (0.23)	1.29 (0.25)	1.17 (0.30)	1.14 (0.31)	84.214 (<0.001)	2.411 (0.121)	0.010 (0.920)
TG	2.07 (1.03)	2.00 (0.84)	2.24 (0.97)	2.23 (1.00)	12.668 (<0.001)	0.589 (0.443)	0.285 (0.593)
LDL-C	2.62 (0.74)	2.69 (0.65)	3.18 (0.87)	3.32 (0.85)	154.913 (<0.001)	4.988 (0.026)	0.494 (0.482)
Female, *n* (%)	350 (65.2%)	98 (69.0%)	468 (62.3%)	213 (74.2%)	0.025 (0.874)	11.537 (0.001)	*N*
Education, *n* (%)	—	—	—	—	2.801 (0.423)	302.224 (<0.001)	*N*
Junior high school	77 (14.3%)	75 (52.8%)	104 (13.8%)	157 (54.7%)	—	—	—
High school	259 (48.2%)	50 (35.2%)	351 (46.7%)	99 (34.5%)	—	—	—
College	169 (31.5%)	15 (10.6%)	242 (32.2%)	23 (8.0%)	—	—	—
Postgraduate	32 (6.0%)	2 (1.4%)	54 (7.2%)	8 (2.8%)	—	—	—
Overweight/obesity (n) (%)	—	—	—	—	70.342 (<0.001)	4.564 (0.102)	*N*
Normal (BMI <24)	277 (51.6%)	75 (52.8%)	257 (34.2%)	82 (28.6%)	—	—	—
Overweight (24 ≤ BMI < 28)	251 (46.7%)	66 (46.5%)	452 (60.2%)	193 (67.2%)	—	—	—
Obesity (BMI ≥ 28)	9 (1.7%)	1 (0.7%)	42 (5.6%)	12 (4.2%)	—	—	—

*Note*: *N*, no variable was not included in the analysis because they were not eligible.

Abbreviations: BMI, body mass index; HAMD, Hamilton Depression Scale; HDL-C, high-density lipoprotein cholesterol; LDL-C, low-density lipoprotein cholesterol; MDD, major depressive disorder; SCH, subclinical hypothyroidism; TC, total cholesterol; TG, triglycerides.

**Table 2:** Relationship between SCH and clinical data in young and middle-aged MDD patients.

Variables	Young		Middle-aged	
	*r*	*p*	*r*	*p*
Duration of illness	0.189	<0.001	0.308	<0.001
Age of onset	0.051	0.068	−0.085	0.077
HAMD	0.421	<0.001	0.333	<0.001
TC	0.463	<0.001	0.453	<0.001
HDL-C	−0.249	<0.001	−0.248	<0.001
TG	0.110	<0.001	0.096	0.047
LDL-C	0.341	<0.001	0.375	<0.001
Gender	−0.029	0.294	0.055	0.257
Education	0.022	0.437	−0.019	0.702
Overweight/obesity	0.185	<0.001	0.245	<0.001

Abbreviations: HAMD, Hamilton Depression Scale; HDL-C, high-density lipoprotein cholesterol; LDL-C, low-density lipoprotein cholesterol; MDD, major depressive disorder; SCH, subclinical hypothyroidism; TC, total cholesterol; TG, triglycerides.

**Table 3:** Demographic and clinical variables independently associated with SCH in each age group.

Variables	Young					Middle-aged				
	*B*	Wald statistic	*p*	OR	95% CI	*B*	Wald statistic	*p*	OR	95% CI
Duration of illness	0.068	15.143	<0.001	1.070	1.034–1.107	0.076	10.298	0.001	1.079	1.030–1.130
HAMD	0.207	50.075	<0.001	1.231	1.162–1.303	0.101	4.380	0.036	1.107	1.006–1.217
TC	0.653	44.452	<0.001	1.921	1.585–2.327	0.634	13.602	<0.001	1.885	1.346–2.641
HDL-C	−1.872	48.741	<0.001	0.154	0.091–0.260	−1.215	7.307	0.007	0.297	0.123–0.716
TG	−0.068	0.901	0.343	0.934	0.812–1.075	*N*	*N*	*N*	*N*	*N*
LDL-C	0.154	2.131	0.144	1.166	0.949–1.433	0.494	6.496	0.011	1.639	1.121–2.398
Overweight (24 ≤ BMI < 28)	0.759	29.356	<0.001	2.135	1.623–2.809	1.210	22.005	<0.001	3.354	2.023–5.561
Obesity (BMI ≥ 28)	1.410	11.404	0.001	4.096	1.807–9.258	3.077	6.453	0.011	21.695	2.020–233.056

*Note: N*, no variable was not included in the analysis because they were not eligible.

Abbreviations: BMI, body mass index; CI, confidence interval; HAMD, Hamilton Depression Scale; HDL-C, high-density lipoprotein cholesterol; LDL-C, low-density lipoprotein cholesterol; OR, odds ratio; SCH, subclinical hypothyroidism; TC, total cholesterol; TG, triglycerides.

## Data Availability

The data that support the findings of this study are available from the corresponding author upon reasonable request. Requests may be sent to the corresponding author: zhangxy@psych.ac.cn.
